# Smoking Is Associated with an Increased Risk for Surgery in Diverticulitis: A Case Control Study

**DOI:** 10.1371/journal.pone.0153871

**Published:** 2016-07-28

**Authors:** Michael J. Diamant, Samuel Schaffer, Stephanie Coward, M. Ellen Kuenzig, James Hubbard, Bertus Eksteen, Steven Heitman, Remo Panaccione, Subrata Ghosh, Gilaad G. Kaplan

**Affiliations:** 1 Department of Medicine, University of Calgary, Calgary, Alberta, Canada; 2 Department of Community Health Sciences, University of Calgary, Calgary, Alberta, Canada; 3 Global Medical Affairs, Shire Canada Inc., Saint-Laurent, Quebec, Canada; The Chinese University of Hong Kong, HONG KONG

## Abstract

**Importance:**

Cigarette smoking increases the risk of surgery in Crohn’s disease. However, the effect of smoking on the need for surgery for diverticulitis is unknown.

**Objective:**

We evaluated whether smoking was a risk factor for surgery among patients admitted to hospital with acute diverticulitis.

**Design:**

We conducted a population-based comparative cohort study of patients admitted to hospital for diverticulitis who were treated with medical versus surgical management.

**Setting & Participants:**

We used the population-based Discharge Abstract Database to identify 176 adults admitted emergently with a diagnosis of diverticulitis between 2009 and 2010 in Calgary.

**Intervention & Main Outcome:**

We performed a medical chart review to confirm the diagnosis of diverticulitis and to extract clinical data. The primary outcome was a partial colectomy during hospitalization. Logistic regression evaluated the association between smoking and surgery after adjusting for potential confounders, including age, sex, comorbidity, and disease severity.

**Results:**

A partial colectomy was performed on 35.6% of patients with diverticulitis and 1.3% died. Among diverticulitis patients, 26.8% were current smokers, 31.5% were ex-smokers, and 41.6% never smoked. Compared to non-smokers, current smokers (adjusted odds ratio [OR] 9.02; 95% confidence interval [CI]: 2.47–32.97) and former smokers (adjusted OR 5.41; 95% CI: 1.54–18.96) had increased odds of surgery.

**Conclusion and Relevance:**

Smoking is associated with the need for surgical management of diverticulitis.

## Background

Diverticulitis is increasing in incidence in North America[1]. The treatment of diverticulitis varies based on disease severity, with milder cases most commonly managed medically and severe, complicated, or life-threatening diverticulitis managed surgically[2]. Complications of diverticulitis include abscess or fistula formation, obstruction, bleeding, or perforation, and occur in approximately 15% to 20% of patients[3, 4]. Between 10% and 45% of patients with diverticulitis require surgery[5, 6], with an estimated 25% of patients requiring surgery during their initial hospital admission for diverticulitis[6].

Age has been shown to be a strong risk factor for the development of diverticulitis and younger age at diagnosis is associated with an increased risk of colectomy [7, 8]. However, regional variability in incidence rates[1] and racial disparities in peri-operative morbidity and mortality[9] suggest that environmental factors may influence the risk of developing diverticulitis and its prognosis.

While previous studies have examined the effect of smoking on the development of diverticulitis and its severity[10–18], the paucity of studies have led to uncertainty regarding smoking’s role in diverticulitis. Further, the effect of smoking on the need for surgery for diveriticulitis is not known. We hypothesize that smoking may also influence the need for surgery for diverticulitis.

The objectives of the study were to determine whether smoking was an independent risk factor for the need for surgical management of diverticulitis.

## Methods

The Discharge Abstract Database (DAD) was received from the Data Integration Management and Reporting (DIMR) administrative healthcare database, which is a population-based administrative database [19]. The DAD includes all hospitalizations in the Calgary Health Zone of Alberta Health Services in Canada, a single-payer, public health authority encompassing approximately 1.3 million people. DAD captures the following characteristics: age, sex, area of residence (delineated from postal code), admission and discharge dates, admission type (emergent, urgent, or elective), transfer to or from hospitals, diagnostic and procedural codes, and hospital charges[23]. Diagnostic and procedural coding is based upon the *International Classification of Disease*, *Tenth Revision*, *Clinical Modification* and the *Canadian Classification of Health Intervention*, respectively. The use of DAD has been previously validated for tracking post-operative complications among ulcerative colitis patients[20].

We used the DAD to identify all patients admitted to hospital for diverticulitis in the Calgary Health Zone between April 1, 2009 and March 31, 2010. Chart review was also used to confirm the diagnosis of diverticulitis, assess whether patients received surgery during their admission, evaluate smoking status at admission, and extract relevant clinical data.

The primary outcome was a partial colectomy for the treatment of diverticulitis. The primary exposure was smoking status at admission to hospital, which was defined as current, former (no use in the past 30 days), or never smoker. Patients with missing data on smoking were excluded from the study population (n = 27). The following covariates were recorded from the medical chart: age at admission to hospital, sex, comorbidities, length of stay, and death in hospital. We assessed disease severity of diverticulitis as defined by Hinchey Classification[21], which categorizes diverticulitis severity as follows: I) localized abscess; II) pelvic abscess; III) purulent peritonitis; and IV) feculent peritonitis.

The Wilcoxon rank sum test was used to compare medians of continuous variables, the Fisher exact test was used to compare nominal categorical variables, and the ordinal categorical variables were compared using Cochran-Mantel-Haenszel row mean score statistics. All risk estimates are represented by adjusted odds ratios (OR) with 95% confidence intervals (CI) created using logistic regression. For all multivariable analyses, the primary outcome was colectomy and the primary exposure was smoking status defined as current, former, or never (referent). The model was adjusted for the following confounders: age, categorized as 18–39, 40–64, and ≥65 years old; sex; comorbidity index score [22], categorized as 0, 1–2, or >2 comorbidities; and Hinchey classification, categorized as II, III, or IV vs 0 and I (referent). A sensitivity analysis was conducted to evaluate the 27 patients with diverticulitis who did not have their smoking status recorded in their chart. For the sensitivity analysis we repeated the preceding regression analysis with the assumption that no record of smoking in the chart meant they were never smokers.

All statistical analyses were conducted using SAS, version 9.3 (SAS Institute Inc., Cary, NC, USA). In all instances an *a priori* α of 0.05 was used. The Conjoint Health Research Ethics Board (CHREB) at the University of Calgary approved the study protocol (Ethics ID: 23976). The CHREB approved a waiver of consent based on Section 50 of the Health Information Act on the grounds that obtaining consent was impractical.

## Results

Among the 149 patients with diverticulitis and smoking status recorded in their chart, 53 (35.6%) underwent partial colectomy and 1.3% of patients died. Table 1 presents the disease characteristics stratified by need for surgery for diverticulitis. Those who required surgery had greater disease severity, more complicated diverticulitis, and longer length of stay as compared to those with medically managed diverticulitis (Table 1).

**Table 1:** Baseline characteristics of patients stratified by surgery and no surgery.

	Total	Surgery	No Surgery	*P*-value
	n = 149	n = 53	n = 96	
**Age** (years)				0.828^β^
Median (IQR)	56 (48–71)	59 (50–69)	56 (47–73)	
**Age groups** (years), n (%)				0.822^α^
18–39	1 (7%)	4 (8%)	7 (7%)	
40–64	87 (58%)	30 (57%)	57 (59%)	
65 and older	51 (34%)	19 (36%)	32 (33%)	
**Length of stay** (days)				< 0.001^β^
Median (IQR)	5 (3–11)	12 (8–18)	4 (3–5)	
**Sex,** n (%)				0.499*
Male	73 (49%)	28 (53%)	45 (47%)	
Female	76 (51%)	25 (47%)	51 (53%)	
**Smoking status,** n (%)				0.006^α^
Current	40 (27%)	18 (34%)	22 (23%)	
Ex-smoker	47 (32%)	22 (42%)	25 (26%)	
Never	62 (42%)	13 (25%)	49 (51%)	
**Comorbidity,** n (%)				0.088^α^
0	71 (48%)	20 (38%)	51 (53%)	
1	34 (23%)	14 (26%)	20 (21%)	
≥ 2	44 (30%)	19 (36%)	25 (26%)	
**Mortality,** n (%)				0.125*
No	147 (99%)	51 (96%)	96 (100%)	
Yes	2 (1%)	2 (4%)	0 (0%)	
**Multiple admissions,** n (%)				0.687^α^
0	130 (87%)	45 (85%)	85 (89%)	
1	13 (9%)	6 (11%)	7 (7%)	
≥ 2	6 (4%)	2 (4%)	4 (4%)	
**Perforation,** n (%)				< 0.001*
No	101 (68%)	23 (43%)	78 (81%)	
Yes	48 (32%)	30 (57%)	18 (19%)	
**Abscess,** n (%)				< 0.001*
No	97 (65%)	20 (38%)	77 (80%)	
Yes	52 (35%)	33 (62%)	19 (20%)	
**Peritonitis,** n (%)				< 0.001*
No	130 (87%)	35 (66%)	95 (99%)	
Yes	19 (13%)	18 (34%)	1 (1%)	
**Hinchey classification,** n (%)				< 0.001^α^
0	89 (60%)	14 (26%)	75 (78%)	
I	30 (20%)	12 (23%)	18 (19%)	
II	8 (5%)	5 (9%)	3 (3%)	
III	17 (11%)	17 (32%)	0	
IV	5 (3%)	5 (9%)	0	

IQR = Interquartile Range

^α^Cochran-Mantel-Haenszel row mean score test.

^β^Wilcoxon rank sum test.

*Fisher’s exact test.

Among diverticulitis patients, 26.8% were current smokers, 31.5% were ex-smokers, and 41.6% never smoked. Compared to non-smokers, current smokers (adjusted OR 9.02, 95% CI: 2.47–32.97) and former smokers (adjusted OR 5.41, 95% CI: 1.54–18.96) had an increased odds of requiring surgery after adjusting for covariates including age (age 40–64 versus 18–39 years: adjusted OR = 0.61, 95% CI: 0.01–3.04) and Hinchey Disease Severity Classification (II, III, or IV versus 0 or I: adjusted OR 71.20, 95% CI: 14.98–338.33) (Table 2). A sensitivity analysis that assumed diverticulitis patients without smoking status recorded in their chart (n = 27) were never smokers yielded attenuated yet significant associations for current smokers (adjusted OR 4.72; 95% CI: 1.66–13.48) and former smokers (adjusted OR 3.39; 95% CI: 1.22–9.47) when compared to never smokers.

**Table 2:** Unadjusted and adjusted odds ratios with 95% confidence intervals (CI) of requiring surgical management of diverticulitis.

	Crude Odds Ratio (95% CI)	Adjusted Odds Ratio (95% CI)
**Smoking**		
Never	1.00	1.00
Former smoker	3.32 (1.33–8.40)	5.41 (1.54–18.96)
Current smoker	3.08 (1.18–8.11)	9.02 (2.47–32.97)
**Age (years)**		
18–39	1.00	1.00
40–64	0.92 (0.21–4.64)	0.61 (0.12–3.04)
>64	1.04 (0.23–5.49)	1.07 (0.18–6.42)
**Sex**		
Female	1.00	1.00
Male	1.27 (0.61–2.63)	0.79 (0.32–1.98)
**Comorbidity**		
0	1.00	1.00
1	1.78 (0.69–4.56)	1.71 (0.56–5.26)
≥ 2	1.94 (0.81–4.59)	1.18 (0.35–4.01)
**Hinchey Disease Severity Classification**		
0, I	1.00	1.00
II, III, IV	32.19 (8.64–173.56)	71.20 (14.98–338.33)

## Discussion

This study demonstrated that current and former smokers were more likely than never smokers to require a partial colectomy to manage their diverticulitis following admission to hospital. By completing a chart review for each diverticulitis admission we confirmed the diagnosis of diverticulitis and the need for surgery. Further, we were able to ascertain smoking status at time of hospital admission assuring that smoking and quitting smoking preceded the admission to hospital for diverticulitis and the need for colectomy in management. Current smokers with diverticulitis had more than nine times the odds of requiring a partial colectomy as compared to never smokers. The strength of this association approximates the magnitude of associations for established risk factors such as smoking and lung cancer[23].

In order to objectively compare our findings to the literature, we conducted a systematic review of observational studies assessing the relationship between smoking and diverticulitis. A systematic literature search of MEDLINE (1950 to May 2, 2013) and EMBASE (Excerta Medica Database; 1980 to May 2, 2013) was conducted to identify studies examining the association between smoking and the risk of developing diverticulitis or the risk of needing surgery for diverticulitis. The included articles are summarized in Table 3. Three studies[12, 13, 15] found statistically significantly higher odds or proportions of patients developing complications of diverticulitis among smokers compared to non-smokers, whereas one study was not significant[14]. Additionally, three studies[11, 16, 18] found statistically significantly higher odds or proportions of developing diverticulitis itself among smokers compared to non-smokers. Also, a case-control study[10] found a higher number of pack-years smoked among patients who received surgical treatment for diverticulitis compared to those who did not, a finding that bordered on statistical significance. Lastly, a longitudinal, observational study found a slightly higher risk of recurrence among diverticulitis patients that smoked compared to those that did not, but this finding did not reach statistical significance [17]. Thus, the findings of our study are consistent with prior studies evaluating the effect of smoking on diverticulitis. Including our study, six of the ten observational studies have shown a statistically significant association between smoking status and diverticulitis development or other prognostic factors.

**Table 3:** Systematic review of observational studies that have studied the relationship between smoking and diverticulitis.

Author, Year (no. of subjects)	Study Design	Study Population	Diverticulitis Definition	Comparator	Outcome	Effect Estimate (95% CI)
Ahmed [18], 2012 (n = 281)	Cohort	Diverticulosis vs. diverticulitis	By CT	Smoking vs. no smoking	% smoking, diverticulosis vs. diverticulitis	33.3% in diverticulitis vs. 21.6% in diverticulosis (p<0.05)
Hall [17], 2011 (n = 672)	Cohort	Diverticulitis	ICD-9 codes	Smoking vs. no smoking	Risk of recurrence	HR 1.17 (0.788–1.748)
Hjern [16], 2011 (n = 35,089)	Cohort	Women with no diagnosis	ICD-10 codes	Current smoker, past smoker, non-smoker	Diagnosis of diverticular disease or diverticulitis	All smokers vs. never, RR 1.32 (1.11–1.57); current vs non-smokers, RR 1.30 (1.06–1.61); past smokers vs. non-smokers, RR 1.34 (1.09–1.64)
Kakarla [15], 2012 (n = 7629)	Cohort	Symptomatic colonic diverticulosis in colectomy patients	Unclear, based on ICD-9 codes	Smoking vs. no smoking	Overall morbidity, serious morbidity, wound complications	Overall morbidity, OR 1.19 (1.03–1.37); serious morbidity, OR 1.34 (1.10–1.64); wound complications, OR 1.25 (1.07–1.46)
Kim [14], 2012 (n = 190)	Cross-sectional	Acute diverticulitis patients	By CT, barium enema, colonoscopy, or pathology	Current smoking vs. no smoking	Severe vs. non-severe diverticulitis (defined as perforation, abscess, phlegmon, fistula, obstruction, sepsis, or peritonitis requiring surgery)	OR 0.60 (0.20–1.78)
Papigrigoriadis[13], 1999 (n = 80)	Prospective cohort	Diverticulitis patients with complications vs. diverticulosis	Not specified	Current smoking vs. no smoking	Complications of diverticulitis, including perforation, abscess, bleeding, and fistulae	OR 2.9 (1.1–7.3)
Turunen [12], 2010 (n = 261)	Cross-sectional with limited follow-up	Symptomatic colonic diverticulosis in colectomy patients	Confirmed by colonoscopy and pathology	Current smoking vs. no smoking	Complication rates	Recurrent diverticulitis, 10.5% vs 3.0% (p = 0.019); pre-op stricture, 25.4% vs 11.1% (p = 0.008); protective stoma, 4.8% vs 0.5% (p = 0.011); NS for other comparisons
Usai [11], 2011 (n = 207)	Case-control	Diverticulosis (n = 150) and acute diverticulitis (n = 57) patients	Clinical findings, colonoscopy, and CT	Current smoking vs. no smoking	Diagnosis of diverticulitis	OR 2.79 (1.30–5.96)
Yoo[10], 2008, (n = 112)	Case-control	Diverticulitis	Not specified	Smoking history in pack-years	Colectomy vs. no colectomy	Mean of 41 smoking pack-years vs 35 pack-years (p = 0.06)

CI, confidence interval; CT, computed tomography; HR, hazard ratio; *ICD-9* (*10*), *International Classification of Diseases*, *Ninth* (*Tenth*) *Revision*; NS, not significant; OR, odds ratio; preop, preoperative; RR, relative risk.

The effect of smoking and gastrointestinal diseases is well established. For example, Crohn’s disease is an analogous condition to diverticulitis that has been shown to be associated with smoking[24,25]. Smoking increases the risk of developing Crohn’s disease and is associated with a worse prognosis including early surgery and postoperative recurrence. Further, the prognosis of patients with Crohn’s disease who quit smoking over time will approximate that of patients who never smoked[26,27].

Several basic science studies have evaluated the mechanisms by which smoking may influence disease in the gastrointestinal tract. Smoking has been shown to reduce mucus production in the colon[28] and can impair endothelial function that can lead to relative mesenteric ischemia[29]. Additionally, smoking can alter the composition and diversity of the intestinal microbiome[30]. However, future studies specific to diverticulitis are necessary to explain the exact mechanism by which smoking may influence diverticulitis.

Observational studies of environmental risk factors are associated with inherent limitations that should be considered in our study[31]. Diverticulitis is a complicated disease with multiple phenotypes of clinical presentation and range of disease severity. Thus, multiple factors may influence the need for surgery. We adjusted the effect of smoking by disease severity; however, residual confounders may have not been evaluated (e.g., exposure to antibiotics and diet) due to lack of data in the medical charts. Also, the proportion of our population that underwent surgery was high (35.6%), suggesting that our study population reflected patients with high disease severity. Supporting this concern was the finding that 66% of our patients were under 65 years of age when they were admitted to hospital for diverticulitis. The young age of onset may have influenced the high rate of partial colectomies. Consequently, our findings are not necessarily generalizable to patients with milder diverticulitis such as patients managed without hospitalization. Further, we were reliant on the accuracy of data recorded in the chart and were not able to assess dose or duration of smoking. Further, approximately 15% of our cohort did not have data on smoking status recorded on their chart. However, our sensitivity analysis that assumed these individuals were non-smokers demonstrated attenuated yet significant associations for current and former smokers. Thus, future studies in different populations of diverticulitis patients are necessary to establish the consistency of smoking’s effects on diverticulitis.

Our observational study demonstrated temporality and a large strength of association. While the number and quality of prior studies is low, most of these studies demonstrated a positive association. Further, the effect of smoking on Crohn’s disease, a disease analogous to diverticulitis, has been established, including multiple basic science studies indicating biological plausibility. Thus, clinicians can use this information to advise their patients against the harm of smoking on diverticulitis. Gastroenterologists can also warn patients with diverticulosis that smoking may increase their risk of surgery if they develop an acute attack of diverticulitis. This data adds to the growing body of literature that highlights the harmful effects of smoking and will strengthen public policy initiatives to reduce smoking in the general population. In conclusion, smoking is an independent risk factor for the need for surgical management of diverticulitis.
